# The vital role of natural history studies in rare disease: insights from X-linked dystonia parkinsonism

**DOI:** 10.1093/braincomms/fcad125

**Published:** 2023-04-18

**Authors:** Ihtsham Haq, Allison Brashear

**Affiliations:** Department of Neurology, Miller School of Medicine, University of Miami, Miami, FL, USA; Department of Neurology, Jacobs School of Medicine and Biomedical Sciences, University at Buffalo, Buffalo, NY, USA

## Abstract

This scientific commentary refers to ‘Establishing a natural history of X-linked dystonia parkinsonism’, by Acuna *et al.* (https://doi.org/10.1093/braincomms/fcad106).

**This scientific commentary refers to ‘Establishing a natural history of X-linked dystonia parkinsonism’, by Acuna** ***et al.*** **(** https://doi.org/10.1093/braincomms/fcad106 **)**

Natural history studies are essential for understanding disease progression, informing clinical trial design, and ultimately guiding the development of effective therapies.^1^ These studies can be complex and labour-intensive, requiring careful selection of assessment protocols, extensive follow-up and fastidious data management and analysis. In their recent article in *Brain Communications*, ‘Establishing a natural history of X-linked dystonia parkinsonism’, Acuna *et al*.^2^ present a detailed natural history study of persons with X-linked dystonia parkinsonism (XDP), a rare neurodegenerative movement disorder affecting men from the Philippines island of Panay.

In this large natural history study of XDP the authors demonstrate the importance of identifying the diagnostic biomarkers, predictors of progression and appropriate clinical outcomes needed to design trials for rare diseases. This is particularly crucial in rare diseases, where the small pool of potential patients makes it difficult to resolve signal-to-noise issues by averaging noise. Moreover, natural history studies of rare diseases, particularly those with monogenetic mechanisms, provide a window into the mechanisms of brain function.

The key findings of the paper confirm the current understanding of XDP as well as providing new insights. The authors demonstrate a rapid appearance of bilateral symptoms relative to diseases like Parkinson’s disease, a correlation between dystonia and bradykinesia, a correlation between age of onset and repeat length, and evidence of cognitive dysfunction as a feature of the disease. Interestingly, repeat size did not correlate with any clinical measures. The study also captured the heterogeneity of progression, with two brothers experiencing an unusually late onset and rapid progression. In addition to the significant contribution to our knowledge of XDP, one of the main implications of the work is its methodology and generalizability to natural history studies of other diseases.

This study of XDP is an illustrative example of the multiple challenges involved in performing natural history studies. Rare diseases may be concentrated in difficult-to-reach populations, making it difficult to assemble patient cohorts large enough to achieve statistical power. Natural history studies ideally provide longitudinal data, and obtaining repeat data from the same cohort multiplies these logistical hurdles as well as adding the new task of maintaining a connection with participants over time.

Although some authors have made good use of technology to perform remote visits, this approach has limitations. This is particularly true in movement disorders in which rigidity is an important variable or when researchers and the patient population are not co-located. This work provides a novel and scalable example of how to overcome these challenges by pursuing a hybrid approach. The authors anchored their video visits by conducting an in-person visit as well performed by local colleagues whose scale assessments were standardized through training, as well as consensus discussions to promote agreement between raters. This approach was made possible by choosing scales commonly used for assessing movement disorders, such as the MDS-UPDRS and BMDRS, which have also been employed for remote visits.^3,4^ There are limitations to this approach that are worth discussing, as noted by the authors. These limitations will be ameliorated or exacerbated by key design choices in recruitment criteria, assessment battery composition and implementation, and study duration. It is worth examining these separately and further:

When selecting recruitment criteria, researchers by definition include and exclude potential subjects. The natural history obtained from the study is representative of the group surveyed, not the disease itself. The match between the two definitions will determine the relevance of the data collected for other researchers. In rare disease research, we recommend broad inclusion criteria to maximize the potential for phenotypic characterization. Researchers should include any participant with the presence of the mutation in question whether symptomatic or asymptomatic. This approach does present practical difficulties, as subjects are not often identified through population-based screens. One strategy to address this is surveying asymptomatic family members. Ideally recruitment should target putatively pre-symptomatic family members who are mutation-positive and follow them over time. However, this will necessarily be a smaller portion of an already small cohort and may miss atypical symptoms unless the family members’ medical records are examined. In this study of XDP the inclusion criteria were men aged ≥18 years displaying symptoms of dystonia and/or parkinsonism and confirmed to have the TAF1 SVA repeat expansion on research testing. While this practical definition allows the characterization of symptomatic patients, it may have missed gene-positive patients without dystonia or parkinsonism. It is worth noting that natural history studies are ideally anchored in a causative gene as this maximizes potential yield. For example, natural history studies of a genetic disease like XDP or LRRK-positive Parkinson’s disease enable researchers to consider any observed phenomena that occur significantly more often in mutation positives than in controls as part of the disease phenotype. In contrast, for syndrome-based natural history studies like clinical Parkinson’s disease, it may be less clear. In the same way, a natural history study of patients infected with mycobacteria tuberculum would be expected to provide a different yield than a natural history study of ‘consumption’. Natural history studies of syndromes can identify those causative mechanisms that lead to more biologically anchored studies, a progression that is necessary to properly identify and trial disease-modifying therapies.

Determining disease onset is critical in patient recruitment for natural history studies as characterizing the disease course over time is a core purpose of such studies. However, patient recall can be unreliable and patients may have different definitions of what constitutes disease onset. For example, a patient with gradual and minimally bothersome parkinsonism who eventually develops speech difficulty may cite the onset of the latter as the start of their disease. The authors of the study addressed this issue by using the technique of entry time realignment, which employed a symptom trajectory model to impute an age of onset. While this approach can address misreporting errors, it is limited by the assessed symptoms and assumes a global rate of decline across all measures collected. Nevertheless the use of entry time realignment to assign a date of onset is an important ancillary measure that we recommend all natural history studies use.

Selecting assessment criteria requires balancing completeness and practicality as well as explication and accuracy. Insufficient criteria may fail to capture the full spectrum of a disease, while adding more measures increases patient and researcher burden, potentially limiting recruitment and retention. The authors identified a minimal battery of 21 measures using principal variables analysis, explaining 67.3% of the variance over the course of the study. There are drawbacks to this approach. Larger samples may show that measures that do not factor significantly into variance do so after all. Measures that are relevant in subgroups may not show their relevance across the entire sample, for example creating a measure set that characterizes slow or rapid progressors poorly. A minimal battery should be monitored to prevent a premature locking in of which observations are considered relevant.

Our own experience with rapid-onset dystonia parkinsonism (RDP) is illustrative.^5^ In RDP the idea of a symptom gradient of dystonic symptoms being typical of the condition, with face more affected than the upper limbs which were in turn more affected than the legs, was considered typical of the condition. When our battery was expanded and added to the original data, bulbar predominance persisted but the presence of a true gradient proved to be highly atypical—present in only 4% of mutation-positive individuals.^5^ Expanded assessments also helped demonstrate the heterogeneity of the adult ATP1A3 cohort, so that RDP is now discussed as a point on the ATP1A3 disease spectrum rather than a separate disease.^6^ It is also crucial to ensure that assessments are accessible to the population in question, and it is a major point that the authors ensured that the testing was performed by bilingual neurologists and in participants’ first language. For these reasons, and to ensure the possibility of exploring all relevant measures, we believe it is crucial to retain the entire data set including source data, for future assessments. As technology advances, means of analysis that were not available at the time of the original study may allow new discovery.

The paper emphasizes the significance of devoting sufficient time and effort to establish a localized, robust and concise assessment battery for rare genetic diseases like XDP. It also suggests that a thorough phenotyping of such diseases can offer insights into more common conditions like dystonia and the parkinsonisms. As we gain more knowledge about the interplay between genes, environment and physiological triggers, studies like this will become increasingly crucial. With the growing attention to and funding for rare diseases, research of this nature will be increasingly important for comprehending the effects of genetic mutations on brain function.^7^
